# Unraveling *Hydra* bioelectrical activity on multielectrode array

**DOI:** 10.3389/fbioe.2025.1736024

**Published:** 2026-02-11

**Authors:** Martina Blasio, Claudia Zenna, Daniela Intartaglia, Giuseppina Tommasini, Giuseppe Coppola, Federica Granata, Angela Tino, Silvia Santillo, Claudia Tortiglione

**Affiliations:** Istituto di Scienze Applicate e Sistemi Intelligenti, Consiglio Nazionale delle Ricerche, Pozzuoli, Italy

**Keywords:** behavioral pattern, extracellular signals, *Hydra vulgaris*, multielectrode array (MEA), signal processing

## Abstract

**Introduction**: Multielectrode array (MEA) technology has emerged as a powerful tool for extracellular recording of electrical activity across a wide range of experimental models, from single cells to organoids. Advanced devices have been developed to monitor and stimulate microscale biological systems enabling precise interrogation of cellular networks and tissue-level electrophysiology. Although these technologies generated promising results, they are not yet widely accessible to neuroscientists and neurobiologists due to limitations in adapting MEAs for whole-organism recordings, in maintaining stable tissue-electrode interfaces, and in decoding the complexity and diversity of bioelectrical signals of intact organisms.

**Methods:** In this study, we demonstrate the feasibility of recording the bioelectrical activity from a whole millimeter-sized organism (*Hydra vulgaris*) using a commercially available multielectrode recording system. Additionally, we introduce a custom MATLAB-based algorithm designed for comprehensive analysis and comparison of small animal model extracellular signals.

**Results:** Two distinct recording configurations were evaluated, each differing in the extent of tissue-electrode coupling area and resulting in variations of the recorded bioelectrical pattern.

**Discussion:** Our findings underline the strict dependency of the recordings from the device architecture and highlight the potential of *Hydra* as a versatile model in bioelectronics, with applications ranging from the development and validation of advanced microengineered devices to fundamental studies on neuronal circuits and neuromodulation.

## 1 Introduction

Neural information processing operates through intricate spatiotemporal patterns generated by electrical and chemical signals, a sophisticated multimodal language that remains challenging to fully decode and modulate with high fidelity. To address these challenges and transcend the limitations of conventional neurotechnologies (Feiner and Dvir, 2018; Li H. et al., 2023), researchers developed innovative strategies to achieve a closer integration between electronic and biological systems. Over the past two decades, advances in microscale technologies and bioelectronics revolutionized neuroengineering, leading to significant innovations in the design and functionality of neural interfaces (Owens and Malliaras, 2010; Chen et al., 2021; Tang et al., 2023). Interfaces based on organic conductive materials offered significant advantages over traditional stiff devices (Liao et al., 2015; Berggren et al., 2019; Berggren and Malliaras, 2019; Berggren et al., 2022). Their low stiffness endows them with soft mechanical properties, providing greater tissue compatibility (O’Connor et al., 2015), minimizing faradaic reactions and inflammatory response (Ghezzi et al., 2011; Green and Abidian, 2015; Carnicer-Lombarte et al., 2021; Kim et al., 2024), and accommodating curved geometries required for comfortable microfluidic platforms (Wang et al., 2021; Liu X. et al., 2024). Crucially, Organic Mixed Ionic-Electronic Conductors (OMIECs) facilitate bidirectional communication between solid-state electronic devices, reliant on electron transport, and native neural tissues, which communicate via ionic bioelectrical signaling, i.e., synaptic and action potentials (Martin and Malliaras, 2016; Kim et al., 2024).

These materials are widely utilized in microelectrode technologies (Ingber, 2022; Tanwar et al., 2022; Hajam and Khan, 2024), increasing the effective surface area, reducing the impedance at the electrode-tissue interface, and facilitating microscale geometric designs.

This versatility has spurred innovation across cellular models, enabling *in vitro* and *in vivo* studies hardly and laboriously achievable using traditional neuronal cultures or brain slices. The planar multielectrode array (MEA) has been proven to be effective in non-invasive and long-term extracellular recordings *in vitro*, supporting investigations into neuronal connectivity in rat primary neurons (James et al., 2004) and human astrocytes (Didier et al., 2020; Kuroda et al., 2023). Three-dimensional (3D) microelectrodes emerged as superior alternatives due to their ability to penetrate cellular layers and establish direct contacts with deeper and healthier cells, thereby significantly improving the signal-to-noise ratio and the recording stability (Abu Shihada et al., 2024).

Innovations in MEA design include flexible three-dimensional (3D) architectures (Choi et al., 2021; Wang et al., 2025) tailored to improve the geometry and topography when interfacing with complex 3D tissues, such as neural organoids and spheroids, and exemplified by flower-shaped MEA, (Martinelli et al., 2004), self-rolled biosensor arrays (Kalmykov et al., 2019; Kalmykov et al., 2021), laminar neurogrids (Li et al., 2019; Le Floch et al., 2022; Li Q et al., 2023), multifunctional mesoscale frameworks (Park et al., 2021) and multi-sensor origami platform (Rahav et al., 2024). These innovations have also been applied to the study of unusual excitable systems [Armada-Moreira et al. (2023) (Supplementary Figure S1)].

Despite these remarkable advances, recording the electrical activity from whole animals remains largely unexplored. Microscale technologies hold promises for correlating *in vivo* neural subcircuit dynamics to behavioral outcomes, as demonstrated by a landmark study carried out by Harris et al. (2010) on the central nervous system of *Lymnaea stagnalis*. By keeping sensory nerves intact while studying the brain, researchers were able to monitor natural-like responses to taste stimuli. This work advances understanding of how neuronal networks integrate sensory information to produce specific behaviors.

Nevertheless, investigating neural dynamics across entire organisms using microscale technologies remains challenging, partly due to the inherent limitations of current model organisms.

The ideal model for such investigations must balance biological and technical criteria: it should be small and easy to handle, with simple anatomy and a transparent body that enables high-quality functional imaging, i.e., calcium activity monitoring. Additionally, such a model should feature evolutionarily conserved pathways to facilitate translational relevance across species, and a streamlined nervous system that orchestrates well-defined behaviors (Gonzales et al., 2020).

Few species meet all these criteria, and even suitable candidates demand innovative engineering solutions. On the other side, great challenges in measuring whole animal electrical activity lie in device engineering. Microfluidic trapping devices can precisely position and non-invasively immobilize the organism, while preserving its natural physiology and behavior, a prerequisite for robust *in vivo* experimentation.

Close interdisciplinary collaboration between biologists, engineers, and physicists is therefore essential in order to create platforms that faithfully capture the electrical signals while maintaining the viability of the organism and the accuracy of the experimental data. Hu et al. (2014) engineered the StyletChip, a microfluidic platform that incorporates suction valves and platinum microelectrodes, designed to immobilize the plant-parasitic nematode *Globodera pallida*. This system enabled high-fidelity recordings, with signal quality comparable to those obtained using a suction glass pipette, of rhythmic stylet thrusting, a bioelectrical behavior essential for host root penetration (Lockery et al., 2012).

Similarly, Liu Z. et al. (2024) developed a hybrid MEA-brain activity mapping (BAM) system for zebrafish (*Danio rerio*) larvae, integrating local field potential recording to calcium imaging, and correlated brain-wide dynamics with sensory processing (Supplementary Figure S2A). In millimeter-sized organisms, such as *Caenorhabditis elegans* and *Hydra vulgaris*, the nano-SPEAR platform overcame movement limitations and used subcellular-scale electrodes to measure and correlate precise electrical pattern to specific behaviors (Lockery et al., 2012; Hu et al., 2014; Dupre and Yuste, 2017; Gonzales et al., 2017; Badhiwala et al., 2018; Gonzales et al., 2020; Liu Z. et al., 2024). The natural transparency of the *Hydra*’s body enabled simultaneous calcium imaging and electrophysiology. This integrated approach made it possible to map the neuronal networks responsible for key behaviors. For example, an ectodermal contraction burst (CB) network was found to underlie longitudinal contractions, while two rhythmic potential (RP) networks (one ectodermal and one endodermal) were found to be active during elongations in response to light and radial contractions (Badhiwala et al., 2018; Badhiwala et al., 2021; Gonzales et al., 2020).

Using a more classical approach, we successfully recorded electrophysiological signals from *Hydra vulgaris* polyps by gently trapping a small portion of tissue within a glass suction microelectrode (Supplementary Figure S2B), (Tommasini et al., 2023). This approach enabled stable recordings of bioelectrical signals and revealed that conjugated semiconductor oligomers modulate *Hydra*’s electrical pattern by accelerating contraction pulse (CP) frequency and selectively targeting specific neuronal circuits (Tommasini et al., 2023; Tommasini et al., 2025). Based on this knowledge and considering the practical advantages of the *Hydra vulgaris* model, our aim was to develop a widely accessible system to record small animal electrical activity, broadening the neurobiologist community studying bioelectricity in a model organism. By employing a commercially available MEA system, originally designed for cells growing in monolayer, and with the aid of a simple custom-made adapter, we recorded *Hydra* electrical activity over long periods, and achieved reliable and reproducible electrical patterns. Importantly, we developed a custom code to analyze spontaneous electrophysiological patterns, featuring contraction bursts, single pulses, and inter-contraction period intervals. While the current standard for behavioral analysis relies on imaging-based tracking of micromovements (Badhiwala et al., 2018), our algorithm provides a complementary approach based on measurable parameters, allowing comparison between various physiological states. Our approach, by integrating electrical and behavioral evidence, will enable to perform functional study on *Hydra* neuronal networks, up to date limited to calcium imaging and depending on genetic transformation, and will also broadly impact on the emerging field of deciphering bioelectricity in small animal models.

## 2 Materials and methods

### 2.1 *Hydra* culture

See Supplementary Material.

### 2.2 Tailoring MEA chambers for *Hydra* bioelectrical recording

Electrophysiological recordings from *Hydra* polyps were performed using the MEA2100-Lite System (Multi Channel Systems, Reutlingen, Germany), controlled by the Multi Channel Experimenter Software. Recordings, consisting of 60 traces (*Channel Number*) per registration, were performed from a single animal and acquired at a sampling rate of 10 kHz. The data were exported as Hierarchical Data Format 5 (HDF5) using the Multi Channel Data Manager (Ver. 1.14.9.22193) software and subsequently analyzed by a custom-made MATLAB (MathWorks, Natick, MA) script (Santillo et al., 2025).

3D MEAs were used to closely interface with the *Hydra* cell layer and achieve a higher spatial electrical resolution. The MEA consisted of 60 titanium nitride electrodes, 100 μm in height designed to limit the movements and improve tissue contact (60-3DMEA250/12/100iR-Ti-gr, Multi Channel Systems MCS GmbH).

**FIGURE 1:**
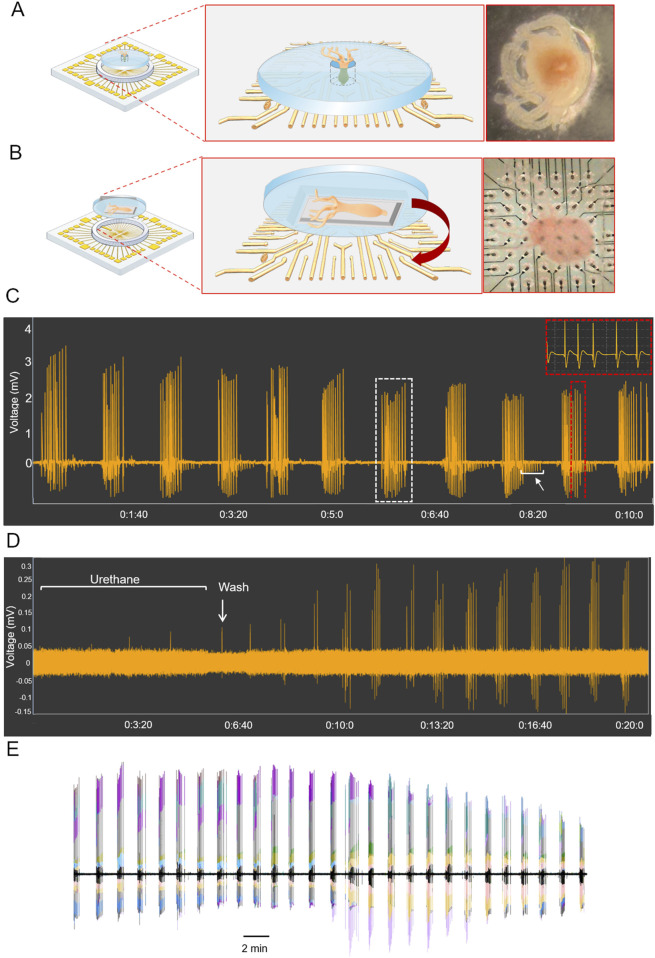
*Hydra vulgaris* on MEA. **(A)** PDMS millimeter-sized channel with the polyp positioned perpendicular (⊥) to the 3D MEA electrodes' area (N = 5); **(B)** PDMS cap adapted to a 3D MEA with the polyp positioned parallel (≑) to the electrodes’ area (N = 5); **(C)** Representative extracellular recording (MEA2100-Lite System) with the insets indicating the CPs (red‐bordered boxes) and the CBs (white‐bordered box). Rhythmic potentials (RPs) are indicated by a white arrow; **(D)** Representative recording of bioelectrical activity of anaesthetized *Hydra* and its recovery after washout. **(E)** Temporal synchrony in signal propagation across sixty channels, each represented by a distinct color.

Recordings were performed with *Hydra* positioned either parallel (≑) or perpendicular (⊥) to the MEA electrodes’ area using two biocompatible Polydimethylsiloxane (PDMS) (Sylgard184, Dow Corning) custom-made caps (Figures 1A,B).

For the parallel configuration, soft lithography replica molding process was employed to create a 2 × 1 mm chamber with a height of 50 μm to gently confine *Hydra* polyp without causing mechanical damage, ensuring extensive electrical coupling while maintaining viability. A silicon wafer was used as photomask substrate, sequentially cleaned with acetone, deionized (DI) water, and isopropanol, and then dried under a nitrogen flow. A negative photoresist ( SU83050 , MicroChem) was spin coated at 3000rpm for 60 s, to reach a target thickness of 50 μm . After baking at 95 °C for 15 min, the photoresist was exposed to UV light via 3D laser writing (MicroWriter ML series system, Durham Magneto Optics) at 250 $mJcm^{-2}$ . The sample was then developed (1 min under gentle agitation, followed by rinsing in DI water and drying under nitrogen flow) to remove the unexposed SU8 and obtain the raised chamber structure. The resulting SU8 mold was then used to obtain the PDMS replica. The base of PDMS and the curing agent were mixed at a 10:1 (w/w) ratio, then degassed under vacuum for 30 min to remove air bubbles and ensure optical transparency. The resulting mixture was subsequently poured onto the prepared SU8 mold, previously treated with Chlorotrimethylsilane ( ${\left(\text{C}\text{H}3\right)}_{3}\text{S}\text{i}\text{C}\text{l}$ ), Sigma-Aldrich) to prevent adhesion. The entire setup was cured on a hot plate at 100 °C for 1h . Thanks to the elastomeric nature of PDMS, the cured replica was easily peeled off the mold without damaging the microchamber.

For perpendicular configuration recordings, we fabricated a PDMS cylinder without using mold to allow faster prototyping. The PDMS mixture was prepared as previously, degassed, and poured directly into a clean silicon wafer and then cured on a hot plate (100 °C, 1 h). Once cured, the PDMS sheet was peeled off and cut into a cylinder ( 0.5cm height, 2.6cm diameter) compatible with the MEA dimension. A central hole ( 1.2mm diameter), aligned with at least two MEA electrodes, was made using a blunt needle to allow the coupling of *Hydra* foot region with MEA electrodes.

### 2.3 A code for *Hydra* biosignals analysis

To enable the accurate and automated detection of peaks in extracellular *Hydra* recordings, we developed a signal processing algorithm, named Hy_CP_Sorting, which exploits the temporal and morphological features of the signal (Santillo et al., 2025). In this section, we provide a formal and mathematical description of its main components. Supplementary Table S1 reports the parameters that define the conditions for classifying and quantifying *Hydra* events, while Supplementary Table S2 describes the typology of characterized events.

Hy_CP_Sorting processes signal data from multiple HDF5 files (q) where q indicates the final number of files. It extracts the time (T) and amplitude (S) vectors from one selected channel (*Channel Number*) of each file, normalizes the amplitude signal with respect to its maximum value (Equation 1), and smooths high-frequency noise using a moving average filter with a window size of 200 samples (*Average Threshold)* (Equation 2)

$$Signal_{norm_{\left(j\right)}}=\frac{Signal_{\left(j\right)}}{max\left(Signal_{\left(j\right)}\right)}$$ (1)

$$Signal_{mean_{\left(i\right)}}=\frac{1}{N}\overset{j+\left(N/2\right)}{\underset{k=j-\left(N/2\right)}{\sum }}Signal_{norm_{\left(j\right)}}$$ (2)

where N =AverageThreshold

For CP peak detection, the algorithm utilizes a numerical method inspired on Taylor series expansion integrating both the slope (first derivative) and concavity (second derivative) signal information. To enhance accuracy, derivatives were calculated using the centered finite difference method for equally spaced data that estimates derivatives using a finite time interval h ( θ/2 in our eqs), defined as the sampled interval between neighboring points, $t_{i}-h$ and $t_{i}+h$ . Then the first derivative, Equation 3, at time $t_{i}$ is estimated as:

$$f^{\prime }\left(t_{i}\right)=\frac{Signal_{\mathrm{mean}}\left(t_{i}+\theta /2\right)-Signal_{\mathrm{mean}}\left(t_{i}-\theta /2\right)}{\left(t_{i}+\theta /2\right)-\left(t_{i}-\theta /2\right)}$$ (3)

where θ=Time Threshold , i.e., the time window used for the derivative.

Similarly, the second derivative, Equation 4, is estimated by applying a finite-difference scheme to already computed first-derivative values, rather than using a direct second-order approximation on the original signal:

$$f^{\prime \prime }\left(t_{i}\right)=\frac{f^{\prime }\left(t_{i}+\theta /2\right)-f^{\prime }\left(t_{i}-\theta /2\right)}{\left(t_{i}+\theta /2\right)-\left(t_{i}-\theta /2\right)}$$ (4)

**FIGURE 2:**
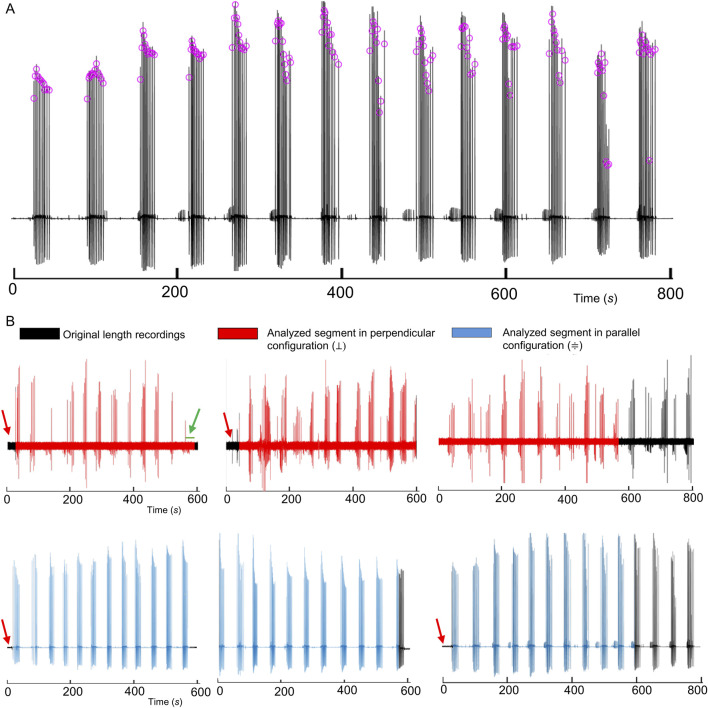
Hy_CP_Sorting Algorithm: Peak Alignment. **(A)** Peaks detection (successive derivative method) in a representative recording (one of ten); **(B)** Six representative recordings (three per condition) in their original length (black traces) overlapped with segments (red and light blue traces) restricted to a time window of 565.519s and used for the comparative analysis between conditions (Santillo et al., 2025). Red arrows mark the pre-peak interval to be removed after peak alignment. The green arrow marks the terminal interval between the last peak and the time window standardization.

Although this procedure may introduce an increased numerical sensitivity compared to classical second-order schemes derived from the Taylor expansion, i.e., may be more sensitive to noise, it resulted, in our context, effective in discriminating between true biological events and transient fluctuations (possibly emphasizing dynamic changes in signal curvature that are critical for identifying genuine peaks, Figure 2A).

Indeed, peaks were identified only if the following conditions were simultaneously satisfied:

- the smoothed signal exceeded the *Normalized Threshold*;
- the first derivative was positive (indicating a rising edge);
- a change in signal concavity, defined by a transition in the second derivative, was observed.

After detecting a valid peak (Equations 3, 4), the algorithm skips ahead by a fixed interval (*Refractory Period*) to avoid detecting redundant or overlapping peaks. Once CPs are identified, all time series are realigned so that the first event is set as zero, effectively discarding the pre-peak interval (red arrow in Figure 2B) and subsequent peak times are re-expressed in the relative form:

$$t_{r,q}=t_{i,q}-t_{1,q}$$

The final length of each recording $\left(t_{\mathrm{final,q}}\right)$ is computed to define a common comparison window $\left(T_{r,q}\right)$ between conditions:

$$T_{r,q}=0\leq t_{r,q}\leq t_{\mathrm{last}}$$

where:

$$t_{\mathrm{last}}=min\left(t_{\mathrm{final,q}}\right)$$

**FIGURE 3:**
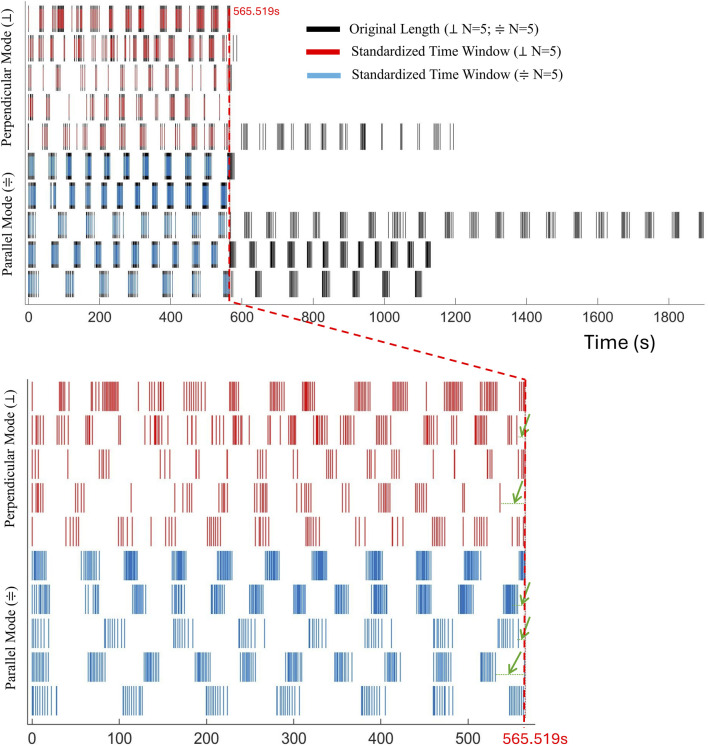
Hy_CP_Sorting Algorithm: Peaks Distribution. Temporal distribution (Raster Plot) of detected peaks both in the original full-length traces (black traces) and in their restricted segments (red and light blue traces). The enlarged inset highlights the events confined in the standardized time window optimized for the statistical analysis (Santillo et al., 2025). Green arrows mark the terminal interval between the last peak and the time window standardization.

In this restricted time window (analyzed segment Figure 2B), the algorithm quantifies and classifies *Hydra* CP events, realigns them in a new matrix ( $T_{p,q}$ , raster plot of Figure 3), and computes inter-peak intervals as:

$$\delta_{p,q}=t_{\left(p+1\right),q}-t_{p,q}$$

while the last interval as:

$$\delta_{p,q}=t_{\left(p_{\mathrm{last}}\right),q}-t_{\mathrm{last}}$$

(green arrow in Figures 2B, 3). Each $\delta_{r,q}$ (CP duration, CPI) is classified as a CP (component of a burst) if $\delta_{r,q}<$ *CP Threshold* or as an IcBI (Intercontraction Burst Interval) if $\delta_{r,q}\geq$ *IcBI Threshold* and in the latter case, the burst counter (nBurst) is incremented by one.

Specifically, Hy_CP_Sorting computes (Supplementary Figure S3; Supplementary Table S2):

- the single CP duration (Contraction pulse interval, CPI), their sum for burst ( ${\text{Burst}}_{\mathrm{Time}}$ ) and their sum for recording file ( ${\text{C}}_{\mathrm{Time}}$ );
- the intercontraction burst duration (IcBI) and their sum ( ${\text{E}}_{\mathrm{Time}}$ );
- the number of bursts (nBurst) and the number of IcBI (nIcBI);
- the number of CP events for each burst ( ${\text{nCP}}_{\mathrm{Burst}}$ ) and recording file (nCP).

In addition, a *Hydra Activity Index* ( ${\text{Hy}}_{\text{AI}}$ ), Equation 5, is calculated by the relationship between the duration of all contraction pulses ( ${\text{C}}_{\mathrm{Time}}$ ) and the duration of all IcBI ( ${\text{E}}_{\mathrm{Time}}$ ).

$$Hy_{\text{AI}}=\frac{\sum_{i=1}^{n}\Delta CP_{i}}{\sum_{j=1}^{n}\Delta IcBI_{j}}\equiv \frac{C_{\mathrm{Time}}}{E_{\mathrm{Time}}}$$ (5)

This index provides a quantitative measure of the state of activity of the animal, reflecting the balance between contractile (time allocated to CP events) and elongation behavior (time allocated to IcBI events).

### 2.4 Statistical analysis

A two-sample *t*-test was performed for statistical significance of mean differences (⊥) *versus* ≑ with * p≤0.05 , while the variance of the main variables was explained by the principal component analysis, PCA (OriginPro2022). All data were represented as box and whisker plots, with the empty square depicting the mean, the thin horizontal bar the median (Q2), the 25^th^ the Q1 , the 75^th^ the Q3 quartile and the whiskers showing the 5^th^ and 95^th^ percentile. Alternatively, data were presented as mean ± standard deviation (SD). The coefficient of variation $\left(\sigma^{*}\right)$ was calculated as the ratio between the standard deviation (σ) and the absolute value of the mean (μ)

$$\sigma^{*}=\frac{\sigma }{\left|\mu \right|}$$

## 3 Results and discussion

The primary objective of this study was to develop a user-friendly tool to perform extracellular electrophysiological recordings in a whole animal, using a compact commercial system, and to establish a method for analyzing its electrical activity. For this purpose, we employed a three-dimensional multielectrode array (3D MEA) to promote optimal contact between electrodes and *Hydra* tissues. Recordings were conducted by positioning polyps in two distinct configurations, either perpendicular (⊥) or parallel (≑) to MEA area, to preserve the spontaneous behavior of the animal while maximizing signal quality. In the perpendicular configuration, each polyp was gently inserted into a millimeter sized vertical channel molded into a PDMS disk, with its foot in contact with one or two microelectrodes (Figure 1A). Alternatively, in the parallel configuration, the polyp is aligned longitudinally along the electrode area and animal movements are spatially confined by a chamber dug in a PDMS cap structure (Figure 1B).

Recordings in both configurations, (≑ N = 5; ⊥ N = 5), revealed a bioelectrical activity pattern consistent with previous studies (Badhiwala et al., 2018; Gonzales et al., 2020; Tommasini et al., 2023; Tommasini et al., 2025). High-amplitude field potentials, named contraction pulses (CPs, Figure 1C, red-bordered box), which are generated by the synchronous bioelectrical activity of large cellular populations, appeared in rhythmic sequences known as contraction bursts (CBs, Figure 1C, white-bordered box). These bursts are typically associated with the activity of longitudinal myofibrils in the outer layer of the epitheliomuscular cells and with *Hydra* full-body contraction behavior (Taddei-Ferretti and Cordella, 1976; Badhiwala et al., 2018).

Low-amplitude electrical events between CBs (Figure 1C, white arrow), identified as rhythmic potentials (RPs) and triggered by contractions of circular myofibrils in the inner tissue layer, were occasionally observed during recordings in parallel configuration suggesting that by improving the tissue-electrode coupling, this recording system reliably enables for studying the bioelectrical behavioral signalling evoked by both ectodermal and endodermal circuits.

The accuracy and sensitivity of this recording system were further confirmed by treating *Hydra* with urethane, a known anesthetic in some invertebrates (Michelson, 1958). As shown in Figure 1D, the anaesthetized polyp exhibited a slow bioelectrical activity, which recovered to a normal frequency after washing (white arrow).

Interestingly, overlaying the sixty traces revealed high synchronous signal propagation across all channels in each recording, although signal amplitudes varied significantly (Figure 1E). This is likely due to the compact micrometric geometry of the MEA, designed to register small events (action potentials), compared to the signal magnitude of a millimeter-sized animal. To address amplitude inconsistencies and optimize events detection, we implemented a code, Hy_CP_Sorting, that identifies and classifies events based on their temporal and morphological features (Supplementary Figure S3), rather than the absolute amplitude (Santillo et al., 2025). Furthermore, to prevent physiological low-amplitude events from being attenuated by averaging traces across all channels and falling below the established peak detection threshold (*Normalized Threshold*), we selected and analyzed the trace with the highest signal-to-noise ratio among the 60 channels, ensuring a consistent identification of all valid electrophysiological events.

After detecting CPs (Equations 3, 4; Figure 2A; see *Materials and Methods*), the analysis code temporally aligned all traces at their first peak, excluding the preceding data (red arrows in Figure 2B). Then, it standardized each segment to the duration of the shortest trace $\left(t_{\mathrm{last}}\right)$ enabling consistent cross-recording comparisons. In our datasets, this procedure allowed the analysis of segments up to 565.519 s.

**FIGURE 4:**
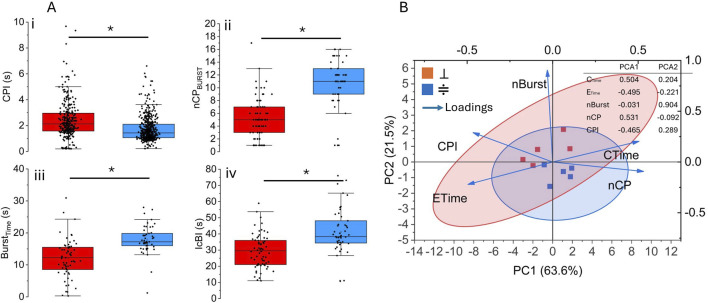
Comparison between perpendicular and parallel conditions. **(A)** Box plots showing the statistical significance of the mean differences between perpendicular (⊥) and parallel (≑) conditions, two sample *t*-test (* p≤0.05 ); **(B)** Biplot of the two principal components (PC1 and PC2), which together explain 85.1% of the total variance (PC1: 63.6%; PC2: 21.5%). The main parameters of both conditions are plotted according to their scores (inset) with the light blue arrows representing their contribution and direction in the PCA space; shaded ellipses denote the confidence intervals for each group.

Six representative traces (black traces) from ten recordings (five per condition for a total of 10 polyps used, Supplementary Figure S2B, Supplementary Tables S4, 5) overlaid with their respective standardized segments (red and light blue traces), while Figure 3 visualizes the raster plot that compares the temporal distribution of detected peaks in all ten recordings, with both the original and the analyzed segments superimposed, and the inset highlighting events detected in the standardized time window. The green arrow (Figures 2B, 3) indicates the terminal interval between the last detected peak and the endpoint defined by the shortest trace ( $t_{\mathrm{last}}$ ), classified either as a CP event or IcBI interval depending on the *IcBI/CP Threshold* criteria. The statistical comparison (two-sample *t*-test, ⊥ vs. ≑, * p≤0.05 ) revealed significant differences with the perpendicular modality (red box) exhibiting a longer mean duration of CP (CPI boxplot, Figure 4Ai) and a lower IcBI (IcBI boxplot, Figure 4Aiv). Despite this significant difference, the average ${\text{BURST}}_{\mathrm{Time}}$ ( ${\text{BURST}}_{\mathrm{Time}}$ boxplot, Figure 4Aiii) and ${\text{C}}_{\mathrm{Time}}$ (Supplementary Tables S3, 4) resulted longer under parallel conditions (light blue box) and with a higher number of CP per burst ( ${\text{nCP}}_{\mathrm{BURST}}$ boxplot, Figure 4Aii). This resulted in a favorable activity index for the parallel conformation (3 out of 5) (Supplementary Tables S4), suggesting infrequent contractions and greater variability in the perpendicular modality. This is further confirmed by the coefficient of variation μ *, (Supplementary Tables S3), and is probably due to the frequent uncoupling of the *Hydra* foot from the electrode surface.

In order to investigate and identify the most informative variables influencing the bioelectrical pattern of the two experimental conditions, we performed the principal components analysis (PCA) on the main *Hydra* parameters, i.e., ${\text{C}}_{\mathrm{Time}}$ , ${\text{E}}_{\mathrm{Time}}$ , nBurst, nCP. The biplot (Figure 4B) reveals a clear segregation between the two experimental conditions, with the principal component 1 (PC1) accounting for 63.6% of the variance. The PC1 was predominantly driven by higher loadings for ${\text{C}}_{\mathrm{Time}}$ , nCP, ${\text{E}}_{\mathrm{Time}}$ and CPI (see inset Figure 4), identifying these parameters as key discriminators between groups. In contrast, PC2 (accounting for 21.5% of the variance), showed a high loading for burst number (nBurst), indicating that this parameter is the dominant source of variability within groups. This pattern is further supported by the non-overlapping confidence ellipses, which reflect the distinct dispersion profiles of the two conditions.

Taken together, these findings indicate that the ${\text{C}}_{\mathrm{time}}$ and the nCP are key discriminators between the two recording configurations. They significantly and positively influence the dynamics of electrical patterns, generating more continuous bursting activity in the parallel configuration, whereas the perpendicular setup exhibits sustained, but less frequent contractions with greater variability resulting from the discontinuos coupling of the foot and electrodes.

In conclusion, the parallel configuration improves signal detection thanks to extensive contact with the MEA, while the microchannel structure better preserves the animal’s natural behavior. These findings highlight the strict dependency of the recordings from the device architecture, suggesting the need for an alternative integrated electronic platform that reconciles these competing requirements. A promising compromise could involve horizontally oriented microchannels housing flattened microelectrodes arranged circularly, longitudinally, or transversely along the channel length. The optimized configuration should provide broad electrical interfacing while preserving the animal’s morphology and behavior, avoiding tissue damage associated with 3D electrodes, and mitigating limitations in spatial resolution. In addition, the integration of suction valves (Hu et al., 2014; Liu Z. et al., 2024) would stabilize animal positioning during long-term electrophysiology recordings, while complementary side channels would facilitate continuous perfusion to prevent medium evaporation and maintain physiological stability. Optical transparency of the custom chip would also enable high-resolution bright-field and fluorescence imaging to correlate electrophysiological signals with functional calcium dynamics. Such a platform, including soft alternatives (Kalmykov et al., 2019; Kalmykov et al., 2021; Li et al., 2019; Li H. et al., 2023; Armada-Moreira et al., 2023), would provide a robust framework to dissect the neural circuits underlying *Hydra*’s behavioral repertoire and establish this animal as a powerful model for neuromodulation and neurotoxicology studies.

## 4 Conclusion

We successfully recorded the bioelectrical activity of *Hydra vulgaris* using the MEA2100-Lite system, a commercially available system, in combination with 3D MEAs adapted to maximize tissue–electrode coupling and allow animal’s natural behavior, which are two key factors for reliable physiological recordings. We developed a custom algorithm for signal processing to detect and classify events in *Hydra*’s bioelectrical pattern. The insights gained from these findings, with their limits, will enable widespread use of the multielectrode technologies among neuroscientists studying bioelectricity in complex systems, where beside the neurons, other cells exhibit electrical activity and contribute to the behavioral patterns. Our results also highlight the crucial contribution of the device configuration on the bioelectrical outcomes, and by identifying the key parameters mainly contributing to the observed signals underpins the development of microfluidic-electronic architectures optimized to balance mechanical confinement and behavioral freedom.

## Data Availability

The original contributions presented in the study are included in the article/Supplementary Material, further inquiries can be directed to the corresponding authors.
