# Supplementary material for: Unraveling Hydra bioelectrical activity on multielectrode array
Source: Front Bioeng Biotechnol. 2026 Feb 11;13:1736024. doi: 10.3389/fbioe.2025.1736024 (PMC12932554; doi:10.3389/fbioe.2025.1736024)
Supplement: Supplementary file 1 [file Supplementaryfile1.pdf]

Supplementary Materials for: Unraveling *Hydra* bioelectrical activity on multielectrode array

This PDF file includes:

- Materials and Methods
- Code Availability
- Figs. S1 to S5
- Tables S1 to S4
- Relative references

1 MATERIALS AND METHODS: *HYDRA* CULTURE

Hydra vulgaris were asexually cultured in *Hydra* Medium (HM) (1 mM $CaCl_2$ and 0.1 mM $NaHCO_3$, pH 7), according to the method descibed by Loomis and Lenhoff (1956). The polyps were fed three times per week with freshly hatched *Artemia salina nauplii* and kept at $18\,^{\circ}\mathrm{C}$ with a $12:12$h light:dark cycle. For all experiments, polyps that had been starved for 24 hours, were selected from a homogeneous population. To anaesthetise the polyps they were incubated in a 2% urethane solution (ethyl carbamate; Sigma, St. Louis, MO) administered before recording. Immediately after treatment, the polyp were placed on the MEA, and recordings were performed both during anaesthesia and after washout.

2 CODE AVAILABILITY

The Hy_CP_Sorting algorithm used to analyze the *Hydra* bioelectrical pattern is deposited in the Zenodo and GitHub repositories (Santillo et al., 2025)

3 SUPPLEMENTARY TABLES AND FIGURES

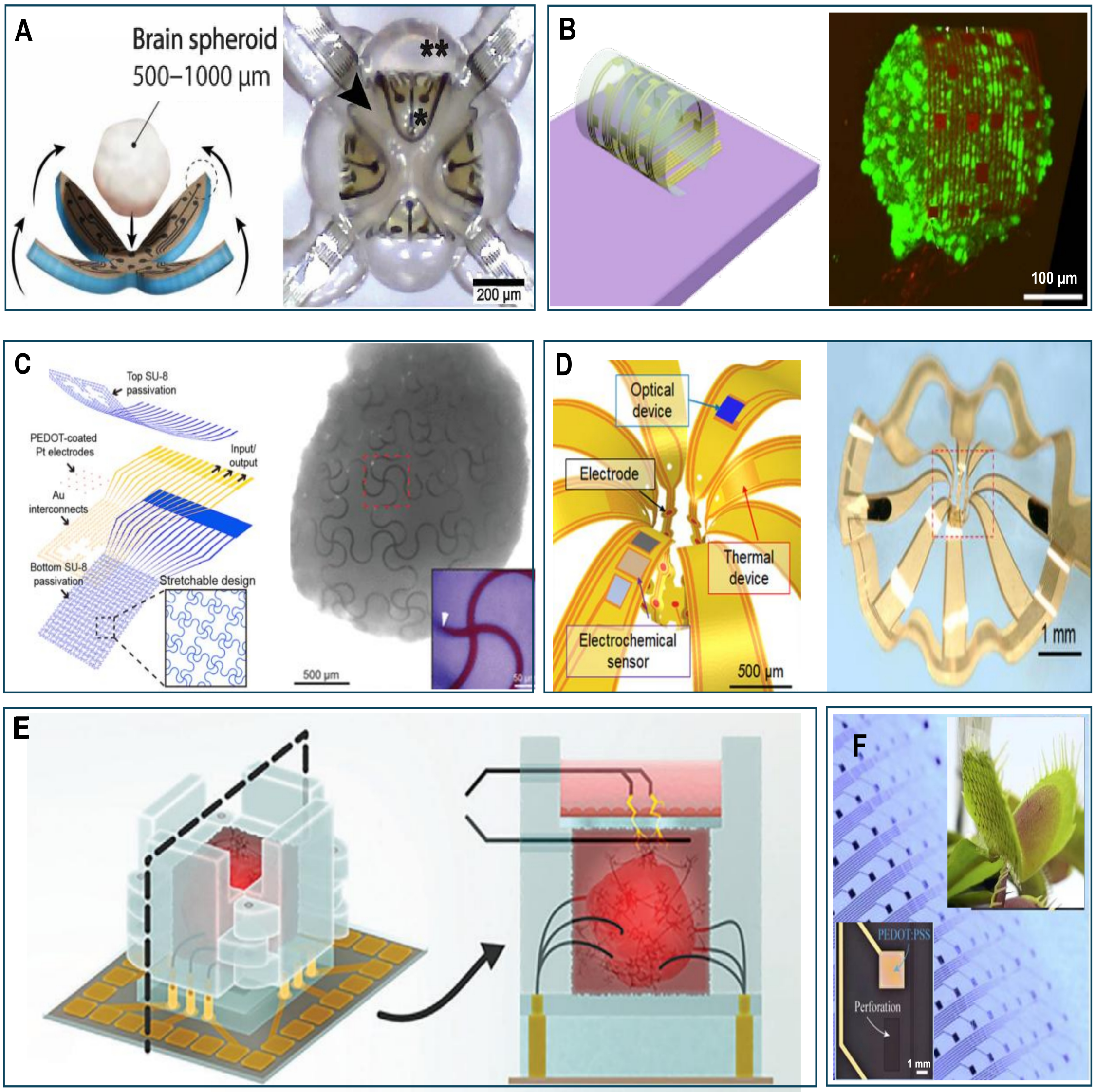

Figure S1: **Flexible bioelectronic platforms.** Schematic illustration and bright field/confocal images of flexible bioelectronic platforms for interaction with organoids and biological tissues. **A)** Flower-shaped MEA wrapping a submillimeter brain spheroid (Martinelli et al., 2024); **B)** Self-rolled biosensor array (3D-SR-BA) for 3D recordings from cortical spheroids (Kalmykov et al., 2019, 2021); **C)** Stretchable laminar neurogrids engineered to have tissue-like properties and adapted for brain organoid development to form *cyborg* systems (Li et al., 2019; Le Floch et al., 2022) integrated with 3D spatial transcriptomics (Li et al., 2023a,b); **D)** Multifunctional mesoscale frameworks (3D MMF), *i.e.* mesostructures, ranging from microns to millimeters, enclose a single neural spheroid centrally and extend eight radially symmetric *wings* each equipped with multifunctional devices (Park et al., 2021); **E)** Sensor arrays embedded in PDMS structures folding around a 3D bioprinted tissue to form a Multisensor Origami Platform, MSOP (Rahav et al., 2024); **F)** MEA NeuroGrid engineered to conform to the curvature of the carnivorous plant *Venus flytrap (Dionaea muscipula)* (Armada-Moreira et al., 2023). All images are reproduced or adapted with permission from the publishers.

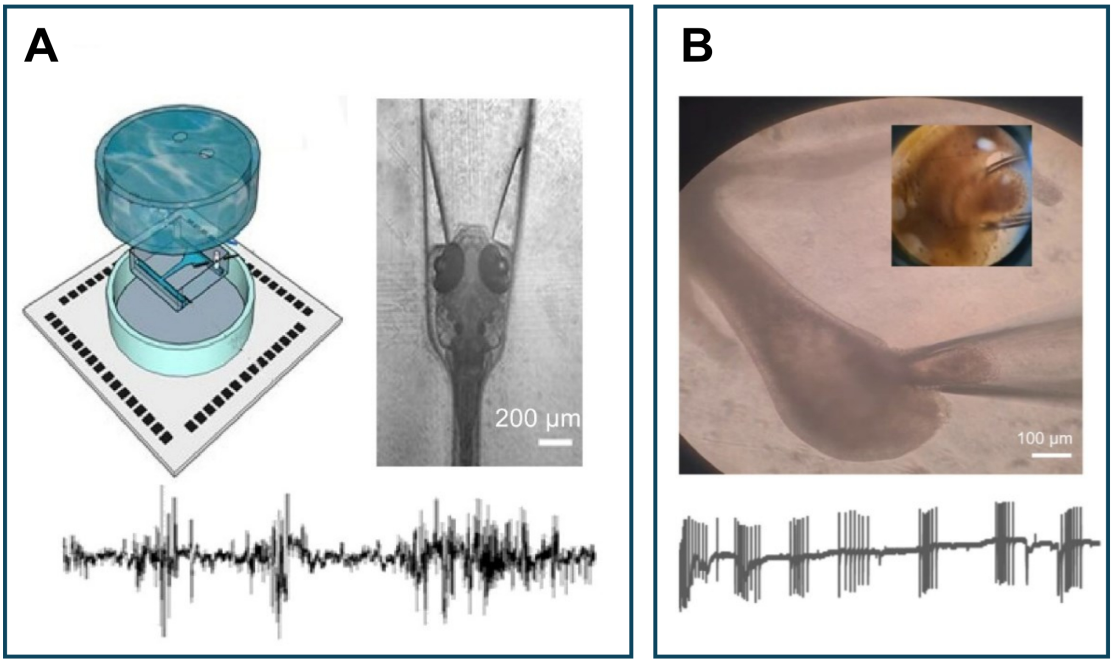

Figure S2: **Different approaches to whole-organism recordings and related electrical signalings. A)** Hybrid MEA-brain activity mapping (BAM) system for *Zebrafish* larvae (Liu et al., 2024); **B)** Suction micropipette sealed to the soft body of *Hydra vulgaris* (Tommasini et al., 2023). All images are reproduced or adapted with permission from the publishers.

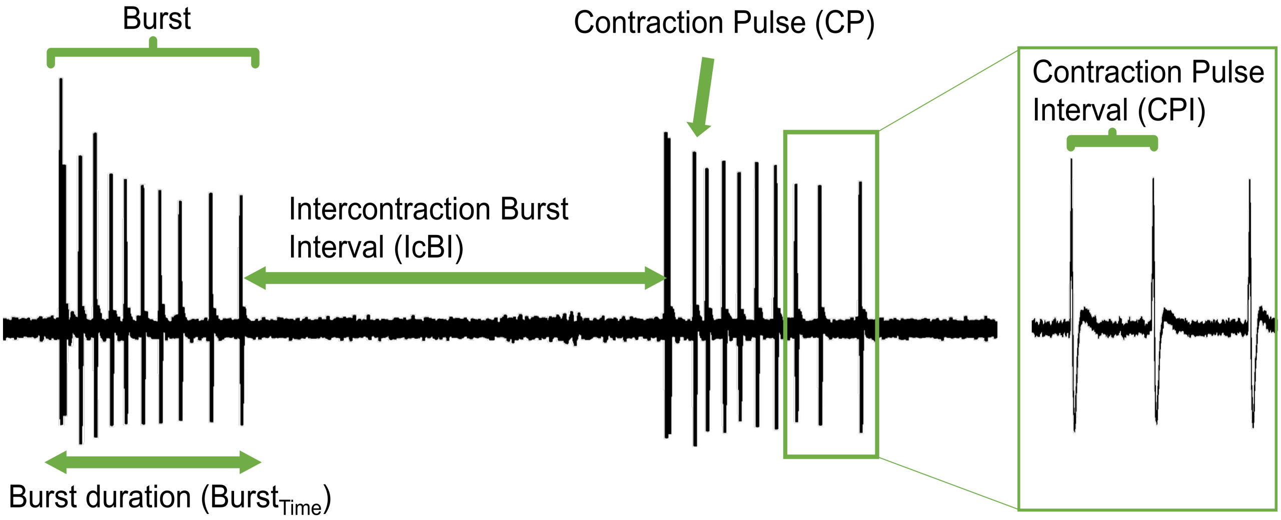

Figure S3: **Mean parameters implemented into the code.** CP: single contraction event; CPI: single CP duration (s); Burst: sequence of CP; Burst$_{Time}$: single burst duration(s); IcBI: interval (s) between burst.

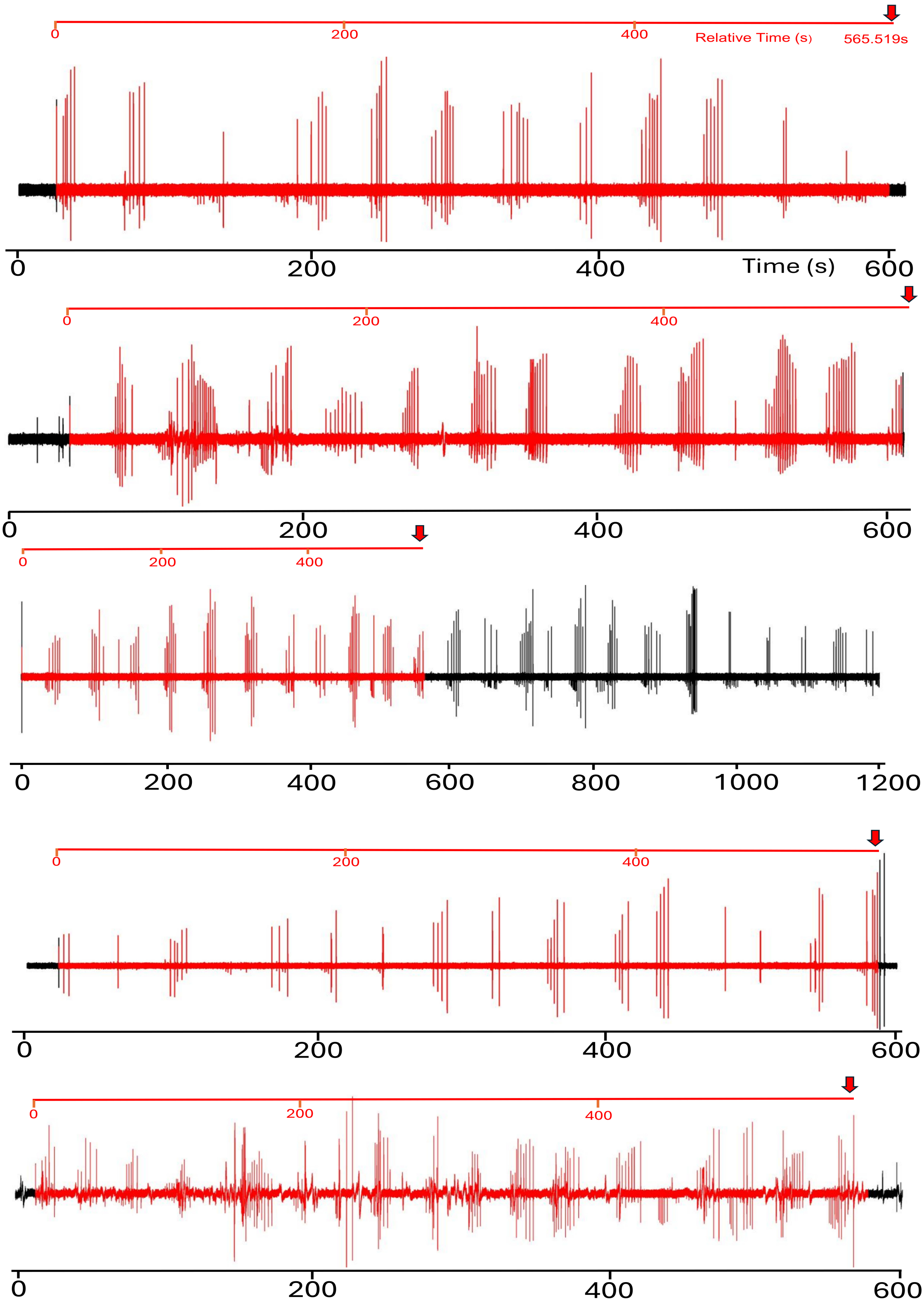

Figure S4: **Recordings with *Hydra* positioned in a perpendicular configuration (⊥) on the MEA.** Original recordings (black traces) with over-imposed (red traces) their respective analyzed segments standardized to the duration of the shortest trace (t_{last}, red arrow).

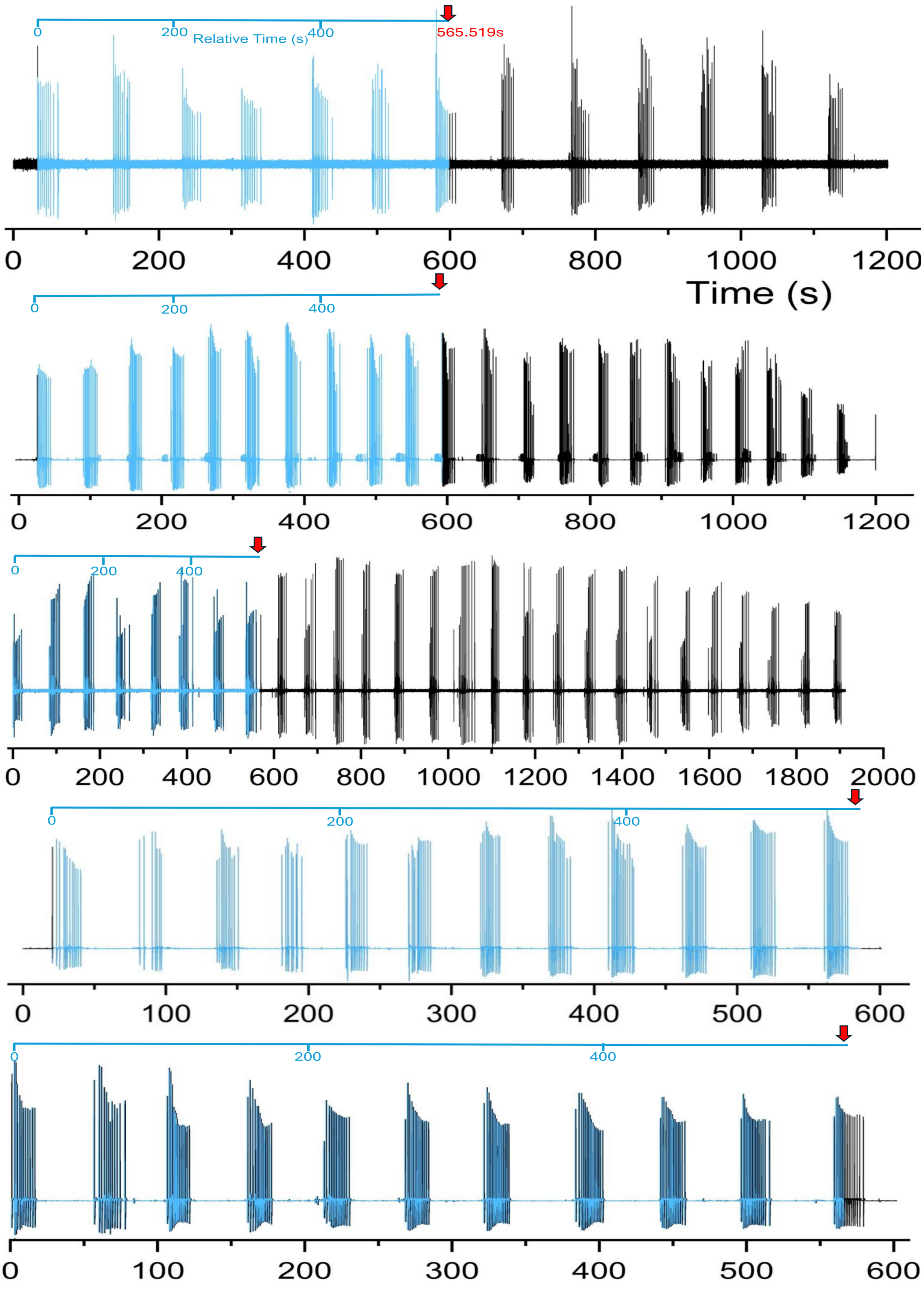

Figure S5: **Recordings with *Hydra* positioned in a parallel configuration (≑) on the MEA.** Original recordings (black traces) with over-imposed (light blue traces) their respective analyzed segments standardized to the duration of the shortest trace (t_{last}, red arrow).

Table S1. The key parameters implemented into the code to classify and quantify electrophysiological events in *Hydra*

**Parameters**	**Description**	**Values**
Channel Number	Index of the signal column in the HDF5 file (1 to 60)	1
Average Threshold	Moving average window length	200
Time Threshold	Sampled interval to estimate the derivative of the signal	10
Normalized Threshold	Percentage of normalized amplitude	0.2
Refractory Period	Minimum time interval (s) between two adjacent peaks	0.2s
IcBI/CP Threshold	Time interval (s) to discern IcBI from CP and bursts	10s

Table S2. *Hydra* events classification

**Parameter**	**Description**	**Parameter**	**Description**
nCP	Total CP number	$\text{Burst}_{\text{Time}}$	Burst duration
$\text{nCP}_{\text{Burst}}$	Intraburst CP number	IcBI	Intercontraction Burst Interval
nBurst	Number of Burst	C_{Time}	Total Contraction Time
nIcBI	Number of IcBI	E_{Time}	Total Elongation Time
CPI	Contraction Pulse Interval	Hy_{AI}	*Hydra* Activity Index

Table S3. Averaged value and variability coefficient (δ*) of electrophysiological parameters for both perpendicular ($\perp$) and parallel ($\div$) configuration

	**Mean ± S.D.**		δ*	
	$\perp$	$\div$	$\perp$	$\div$
CPI	2.42 ± 1.39	1.71 ± 0.94	0.57	0.55
nCP_{Burst}	5.28 ± 3.22	10.84 ±3.73	0.61	0.34
Burst_{Time}	12.05 ± 5.96	17.87 ± 4.46	0.49	0.25
IcBI	29.43 ± 10.26	41.26 ± 14.28	0.35	0.35
C_{Time}	151.86 ± 53.87	175.13 ± 8.69	0.35	0.05
E_{Time}	400.36 ± 55.57	387.87 ± 13.26	0.14	0.03

Table S4. Contraction and elongation time and relative Hy_{AI} for both perpendicular ($\perp$) and parallel ($\div$) configuration

	C_{Time} (s)		E_{Time} (s)		Hy_{AI}	
	$\perp$	$\div$	$\perp$	$\div$	$\perp$	$\div$
Hy1	150.18	168.48	409.45	397.59	0.36	0.42
Hy2	112.01	180.65	420.07	383.63	0.26	0.47
Hy3	86.42	164.32	476.98	402.21	0.18	0.47
Hy4	208.44	185.57	362.62	368.25	0.57	0.50
Hy5	202.23	176.60	332.64	387.67	0.60	0.45

REFERENCES

Armada-Moreira, A., Dar, A. M., Zhao, Z., Cea, C., Gelinas, J., Berggren, M., et al. (2023). Plant electrophysiology with conformable organic electronics: Deciphering the propagation of venus flytrap action potentials. *Science Advances* 9, eadh4443. doi:10.1126/sciadv.adh4443

Kalmykov, A., Huang, C., Bliley, J., Shiwarski, D., Tashman, J., Abdullah, A., et al. (2019). Organ-on-e-chip: Three-dimensional self-rolled biosensor array for electrical interrogations of human electrogenic spheroids. *Science Advances* 5, eaax0729. doi:10.1126/sciadv.aax0729

Kalmykov, A., Reddy, J. W., Bedoyan, E., Wang, Y., Garg, R., Rastogi, S. K., et al. (2021). Bioelectrical interfaces with cortical spheroids in three-dimensions. *Journal of Neural Engineering* 18, 055005. doi:10.1088/1741-2552/abf290

Le Floch, P., Li, Q., Lin, Z., Zhao, S., Liu, R., Tasnim, K., et al. (2022). Stretchable mesh nanoelectronics for 3d single-cell chronic electrophysiology from developing brain organoids. *Advanced Materials* 34, 2106829. doi:https://doi.org/10.1002/adma.202106829

Li, H., Wang, J., and Fang, Y. (2023a). Recent developments in multifunctional neural probes for simultaneous neural recording and modulation. *Microsyst. Nanoeng.* 9. doi:https://doi.org/10.1038/s41378-022-00444-5

Li, Q., Lin, Z., Liu, R., Tang, X., Huang, J., He, Y., et al. (2023b). Multimodal charting of molecular and functional cell states via in situ electro-sequencing. *Cell* 186, 2002–2017.e21. doi:https://doi.org/10.1016/j.cell.2023.03.023

Li, Q., Nan, K., Floch, P. L., Lin, Z., Sheng, H., Blum, T. S., et al. (2019). Cyborg organoids: Implantation of nanoelectronics via organogenesis for tissue-wide electrophysiology. *Nano Letters* 19, 5781–5789. doi:10.1021/acs.nanolett.9b02512. PMID: 31347851

Liu, X., Gong, Y., Jiang, Z., Stevens, T., and Li, W. (2024). Flexible high-density microelectrode arrays for closed-loop brain–machine interfaces: a review. *Frontiers in Neuroscience* 18. doi:10.3389/fnins.2024.1348434

Loomis, W. F. and Lenhoff, H. M. (1956). Growth and sexual differentiation of hydra in mass culture. *Journal of Experimental Zoology* 132, 555–573. doi:https://doi.org/10.1002/jez.1401320309

Martinelli, E., Akouissi, O., Liebi, L., Furfaro, I., Maulà, D., Savoia, N., et al. (2024). The e-flower: A hydrogel-actuated 3d mea for brain spheroid electrophysiology. *Science Advances* 10, eadp8054. doi:10.1126/sciadv.adp8054

Park, Y., Franz, C. K., Ryu, H., Luan, H., Cotton, K. Y., Kim, J. U., et al. (2021). Three-dimensional, multifunctional neural interfaces for cortical spheroids and engineered assembloids. *Science Advances* 7, eabf9153. doi:10.1126/sciadv.abf9153

Rahav, N., Marrero, D., Soffer, A., Glickman, E., Beldjilali-Labro, M., Yaffe, Y., et al. (2024). Multi-sensor origami platform: A customizable system for obtaining spatiotemporally precise functional readouts in 3d models. *Advanced Science* 11, 2305555. doi:https://doi.org/10.1002/advs.202305555

Santillo, S., Blasio, M., Tommasini, G., and Tortiglione, C. (2025). Decoding hydra bioelectrical activity by microelectrode array doi:10.5281/zenodo.17425421

Tommasini, G., Simone, M. D., Santillo, S., Dufil, G., Iencharelli, M., Mantione, D., et al. (2023). In vivo neuromodulation of animal behavior with organic semiconducting oligomers. *Science Advances* 9, eadi5488. doi:10.1126/sciadv.adi5488
